# CYP79 P450 monooxygenases in gymnosperms: CYP79A118 is associated with the formation of taxiphyllin in *Taxus baccata*

**DOI:** 10.1007/s11103-017-0646-0

**Published:** 2017-08-09

**Authors:** Katrin Luck, Qidong Jia, Meret Huber, Vinzenz Handrick, Gane Ka-Shu Wong, David R. Nelson, Feng Chen, Jonathan Gershenzon, Tobias G. Köllner

**Affiliations:** 1 Max Planck Institute for Chemical Ecology, Hans-Knöll-Strasse 8, 07745 Jena, Germany; 2 Graduate School of Genome Science and Technology, University of Tennessee, Knoxville, TN 37996 USA; 3 Present Address: John Innes Centre, Norwich Research Park, Colney Ln, Norwich, NR4 7UH UK; 4 Department of Biological Sciences, University of Alberta, Edmonton, AB T6G 2E9 Canada; 5 Department of Medicine, University of Alberta, Edmonton, AB T6G 2E1 Canada; 6 BGI-Shenzhen, Beishan Industrial Zone, Yantian District, Shenzhen, 518083 China; 7 Department of Microbiology, Immunology, and Biochemistry, University of Tennessee Health Science Center, Memphis, TN 38163 USA; 8 Department of Plant Sciences, University of Tennessee, Knoxville, TN 37996 USA

**Keywords:** *Taxus baccata*, Cytochrome P450 monooxygenase, CYP79, Aldoxime, Cyanogenic glycoside, Conifers, Taxiphyllin

## Abstract

**Key message:**

Conifers contain P450 enzymes from the CYP79 family that are involved in cyanogenic glycoside biosynthesis.

**Abstract:**

Cyanogenic glycosides are secondary plant compounds that are widespread in the plant kingdom. Their biosynthesis starts with the conversion of aromatic or aliphatic amino acids into their respective aldoximes, catalysed by N-hydroxylating cytochrome P450 monooxygenases (CYP) of the CYP79 family. While CYP79s are well known in angiosperms, their occurrence in gymnosperms and other plant divisions containing cyanogenic glycoside-producing plants has not been reported so far. We screened the transcriptomes of 72 conifer species to identify putative *CYP79* genes in this plant division. From the seven resulting full-length genes, *CYP79A118* from European yew (*Taxus baccata*) was chosen for further characterization. Recombinant CYP79A118 produced in yeast was able to convert l-tyrosine, l-tryptophan, and l-phenylalanine into *p*-hydroxyphenylacetaldoxime, indole-3-acetaldoxime, and phenylacetaldoxime, respectively. However, the kinetic parameters of the enzyme and transient expression of *CYP79A118* in *Nicotiana benthamiana* indicate that l-tyrosine is the preferred substrate in vivo. Consistent with these findings, taxiphyllin, which is derived from l-tyrosine, was the only cyanogenic glycoside found in the different organs of *T. baccata*. Taxiphyllin showed highest accumulation in leaves and twigs, moderate accumulation in roots, and only trace accumulation in seeds and the aril. Quantitative real-time PCR revealed that *CYP79A118* was expressed in plant organs rich in taxiphyllin. Our data show that CYP79s represent an ancient family of plant P450s that evolved prior to the separation of gymnosperms and angiosperms. CYP79A118 from *T. baccata* has typical CYP79 properties and its substrate specificity and spatial gene expression pattern suggest that the enzyme contributes to the formation of taxiphyllin in this plant species.

**Electronic supplementary material:**

The online version of this article (doi:10.1007/s11103-017-0646-0) contains supplementary material, which is available to authorized users.

## Introduction

Plants produce a plethora of so called secondary or specialized compounds to defend themselves against a multitude of biotic and abiotic stresses. Many of these compounds are widespread in the plant kingdom while others are specific for a certain plant family or even a single genus or species. Flavonoids and terpenes, for instance, are produced by many plants including angiosperms, gymnosperms, ferns, and mosses (Rausher 2006; Gershenzon and Croteau 1991). In contrast, glucosinolates and benzoxazinoids are only known in angiosperms and are mainly restricted to the Brassicales and Poaceae, respectively (Halkier and Gershenzon 2006; Frey et al. 2009).

Cyanogenic glycosides represent a diverse group of secondary compounds that is widespread in the plant kingdom. So far they have been found in the angiosperms, the conifers, and ferns (Hegenauer 1959; Kofod and Eyjolfsson 1969; Harper et al. 1976; Zagrobelny et al. 2008). Cyanogenic glycosides are derived from aliphatic or aromatic α-hydroxynitriles and are usually stored in the vacuole or other specialized vesicle-like structures (Gleadow and Moller 2014). After tissue disruption caused, for example, by chewing herbivores, cyanogenic glycosides come in contact with β-glucosidases that catalyse the hydrolysis of the β-glycosidic bond between the sugar and the hydroxynitrile moiety. The released α-hydroxynitriles are unstable and can rapidly convert to toxic hydrogen cyanide and highly reactive aldehydes or ketones (recently reviewed in Gleadow and Moller 2014). Cyanogenic glycosides are effective deterrents to generalist herbivores (Gleadow and Woodrow 2002; Zagrobelny et al. 2004) and have been described as defence compounds against pathogens (Osbourn 1996). Moreover, they are discussed as carbon and nitrogen storage and transport forms and as modulators of oxidative stress (Selmar et al. 1988; Moller 2010a).

The biosynthesis of cyanogenic glycosides was first elucidated in *Sorghum bicolor* (McFarlane et al. 1975; Moller and Conn 1979; Halkier et al. 1989) and the enzymes involved have now been identified and characterized in several plant species including sorghum (*S. bicolor*) (Sibbesen et al. 1995; Bak et al. 1998; Jones et al. 1999), the legume *Lotus japonicus* (Takos et al. 2011), and cassava (*Manihot esculenta*) (Andersen et al. 2000; Jorgensen et al. 2011; Kannangara et al. 2011). The first and rate-limiting step of the pathway is catalysed by cytochrome P450 monooxygenases (CYP) of the CYP79 family. CYP79 enzymes accept aromatic and aliphatic amino acids as substrates and convert them into aldoximes by two successive N-hydroxylations, a dehydration and a decarboxylation reaction (Halkier et al. 1995; Sibbesen et al. 1995). The aldoximes formed can be further metabolized by CYP71 or CYP736 enzymes to labile α-hydroxynitriles that are stabilized by rapid glycosylation through the action of family 1 UDP-glycosyltransferases (UGT) (Bak et al. 1998; Jones et al. 1999; Takos et al. 2011; Kannangara et al. 2011).

Although the enzymes involved in cyanogenic glycoside formation have been extensively investigated in the angiosperms, little is known about their occurrence and biological roles in other plant lineages. Multiple conifer species such as European yew (*Taxus baccata*), prickly juniper (*Juniperus oxycedrus*), and dawn redwood (*Metasequoia glyptostroboides*) have been reported to produce taxiphyllin, a tyrosine-derived cyanogenic glycoside (Bijl-van Dijk et al. 1974). Thus, it is likely that these species possess an enzyme similar to angiosperm CYP79s that can convert l-tyrosine into the taxiphyllin precursor *p*-hydroxyphenylacetaldoxime. In this study we screened the transcriptomes from 72 conifer species to search for *CYP79* genes in this plant division. One of the identified candidates, *CYP79A118* from *T. baccata*, was chosen for heterologous expression and in vitro biochemical characterization. In addition, the transcript abundance of *CYP79A118* and accumulation of the cyanogenic glycoside taxiphyllin were measured in the different organs of *T. baccata* trees. Based on our data we conclude that CYP79A118 most likely contributes to the formation of taxiphyllin in *T. baccata*.

## Results

### Identification of CYP79 genes in the conifers

**Fig. 1:**
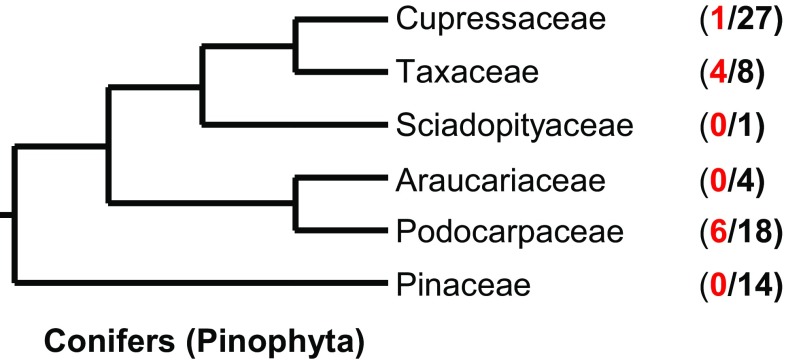
Family distribution of *CYP79* genes identified from the transcriptomes of 72 conifer species. The numbers in parentheses represent the number of transcriptomes containing putative *CYP79* genes (in *red*) and total transcriptomes analysed in each family (in *black*). The phylogeny of the conifers presented was adapted from (Gernandt et al. 2011)

**Fig. 2:**
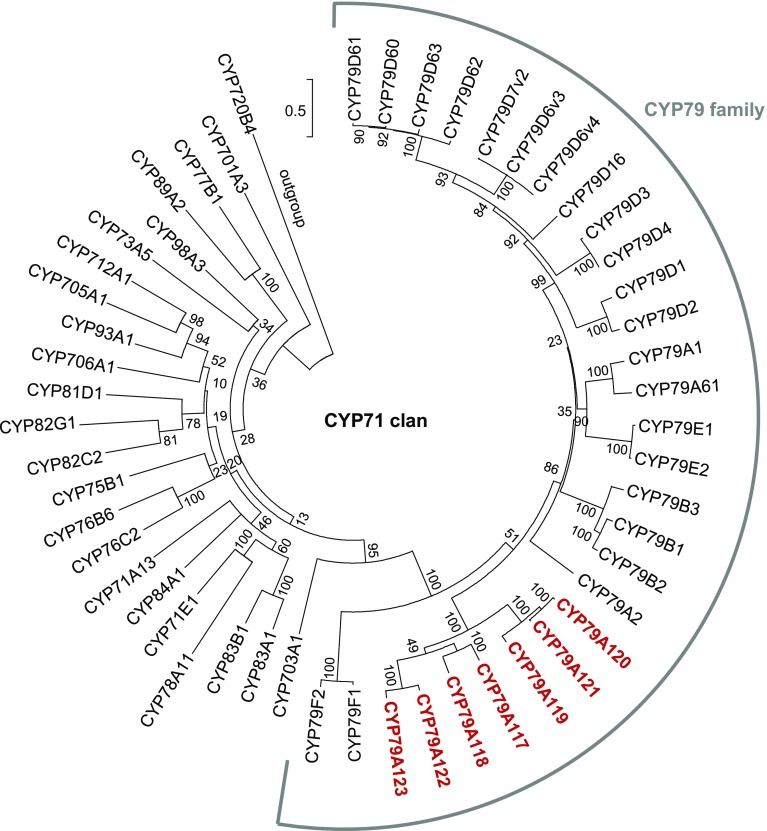
Dendrogram analysis (rooted tree) of conifer CYP79 proteins with characterized CYP79 proteins from angiosperms and selected members of other CYP families belonging to the CYP71 clan (which includes CYP79s). The tree was inferred using the neighbor-joining method and n = 1000 replicates for bootstrapping. Bootstrap values >50 are shown next to each node. The tree is drawn to scale, with branch lengths measured in the number of amino acid substitutions per site. AtCYP710A1 from Arabidopsis was chosen as outgroup

To identify putative *CYP79* genes in the conifers (Pinophyta), transcriptomes from 72 conifer species (Table S1) generated from the OneKP initiative (https://sites.google.com/a/ualberta.ca/onekp/) were screened using a T BLASTN search with four CYP79 proteins (poplar CYP79D6, *Arabidopsis* CYP79F1, maize GRMZM2G011156T01 and GRMZM2G138248T01) from different plant species as queries. At the 38% sequence identity and 10^−20^ E-value cutoff, this initial search resulted in more than 2000 putative sequences. Further phylogenetic analysis with P450 sequences from different CYP families revealed seven full-length genes and ten gene fragments with similarity to angiosperm *CYP79* genes (Fig. 1; Table S2). As currently treated, the conifers comprise six families: Cupressaceae, Taxaceae, Sciadopityaceae, Araucariaceae, Podocarpaceae, and Pinaceae (Gernandt et al. 2011). While seven out of 18 analysed transcriptomes from the Podocarpaceae, five out of eight analysed transcriptomes from the Taxaceae, and one out of 27 analysed transcriptomes from the Cupressaceae contained at least one *CYP79* gene, no sequences with similarity to *CYP79*s were found in the transcriptomes from the Pinaceae, Araucariaceae, and Sciadopityaceae (Fig. 1). A dendrogram analysis of the conifer *CYP79* genes and selected P450 genes from the CYP71 clan showed that the newly identified conifer genes formed a well-defined clade within the CYP79 family (Fig. 2). According to the standard P450 classification system that defines families and subfamilies based on a certain level of amino acid sequence identity (family, >40% identity; subfamily, >55% identity; (Nelson et al. 1996)), the conifer CYP79s were grouped into the CYP79A subfamily (See Table S2). Sequence motifs conserved in A-type P450 proteins such as the heme binding site (consensus sequence ProPheGlyxGlyArgArgxCysxGly) and the ProGluArgPhe motif (Durst and Nelson 1995) were also found in the conifer CYP79 proteins (Fig. 2).

### Biochemical characterization of CYP79A118 from *Taxus baccata*

**Fig. 3:**
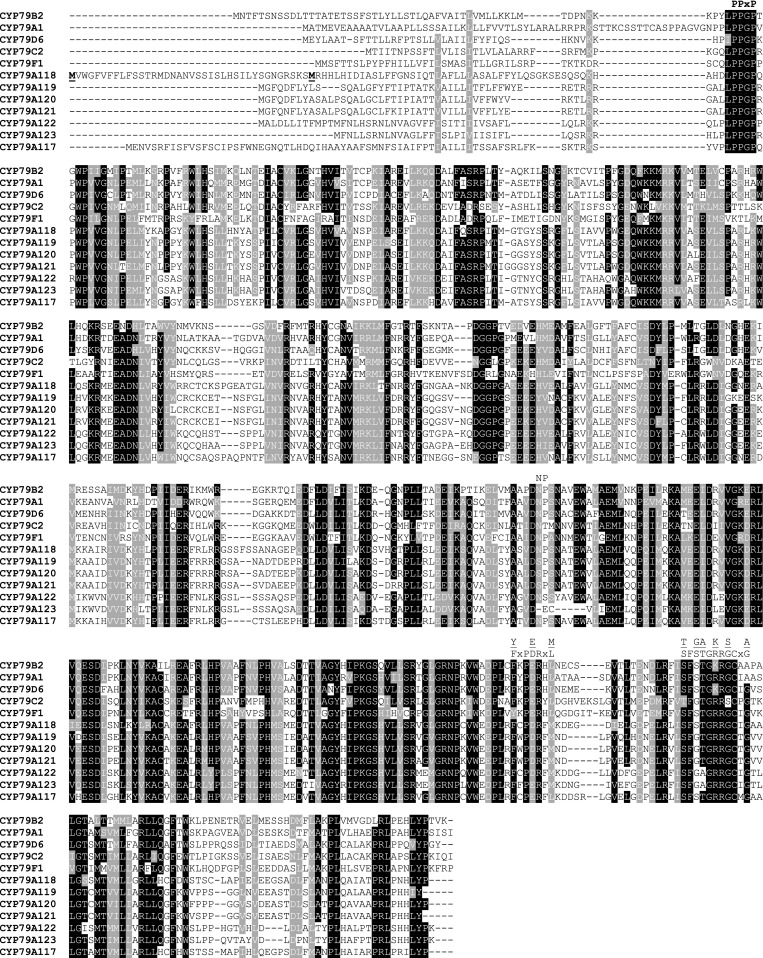
Amino acid alignment of conifer CYP79 enzymes with selected CYP79s from angiosperms. Amino acids identical in at least ten out of 12 sequences are marked by *black boxes* and amino acids with similar side chains are marked by *gray boxes*. Sequence motifs characteristic for CYP79 proteins are labeled. The methionine residue used as start codon for N-terminal truncated CYP79A118-M39 is marked in bold and underlined. CYP79B2, *Arabidopsis thaliana*; CYP79A1, *S. bicolor*; CYP79D6, *Populus trichocarpa*; CYP79C2, *A. thaliana*; CYP79F1, *A. thaliana*

**Fig. 4:**
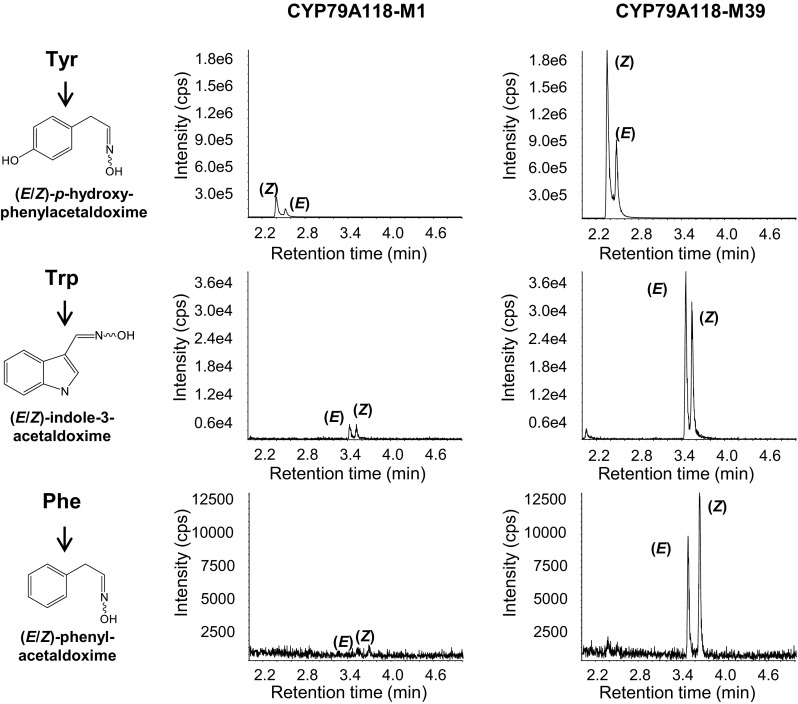
Enzymatic activity of full-length (CYP79A118-M1) and N-terminal truncated (CYP79A118-M39) CYP79A118. The genes were heterologously expressed in *S. cerevisiae* and microsomes containing the recombinant proteins were incubated with the amino acid substrates l-tyrosine (Tyr), l-tryptophan (Trp), and l-phenylalanine (Phe). The aldoximes produced, *p*-hydroxyphenylacetaldoxime, indole-3-acetaldoxime, and phenylacetaldoxime, respectively, were detected using LC-MS/MS. CPS, counts per second (electron multiplier)

For biochemical characterization of conifer CYP79s, we chose the gene WWSS98821 from *Taxus baccata* and expressed it heterologously in *Saccharomyces cerevisiae* strain WAT11, which carries the *Arabidopsis* cytochrome P450 reductase 1 (CPR1) (Pompon et al. 1996). WWSS98821 was designated as CYP79A118 according to the standard P450 nomenclature. Since CYP79A118 contains an extended N-terminus in comparison to angiosperm CYP79s (Fig. 3), the full-length protein and an N-terminal truncated version that starts with methionine at position 39 (CYP79A118-M39; Fig. 2) were both characterized. Microsomes harboring the recombinant proteins and CPR1 were incubated with the potential amino acid substrates l-tyrosine, l-tryptophan, l-phenylalanine, l-leucine, and l-isoleucine in the presence of the electron donor NADPH. Both, the full-length protein as well as the truncated version showed enzymatic activity with the aromatic amino acids and produced p-hydroxyphenylacetaldoxime, indole-3-acetaldoxime, and phenylacetaldoxime, but were not active with l-leucine and l-isoleucine (Fig. 4). Enzyme assays containing microsomes from a yeast culture expressing an empty vector revealed no aldoxime-producing activity and assays performed in the absence of NADPH showed only trace activity (See Fig. S1). Notably, the N-terminal truncated version CYP79A118-M39 had higher enzyme activity than the full-length protein (Fig. 4). However, quantification of the recombinant protein in the yeast microsomes by carbon monoxide difference spectra was inconclusive, and therefore it is still unclear whether the extended N-terminus of CYP79A118 decreased the overall catalytic efficiency of the enzyme or whether it affected the rate of heterologous expression and thus the amount of recombinant protein obtained. Since the substrate specificities of the full-length and truncated CYP79A118 were nearly identical (Fig. 4), we used the more active truncated version for kinetic analysis. The substrate affinity of CYP79A118-M39 for l-tyrosine (*K*_m_, 0.456 ± 0.061 mM) was in the range reported for other previously characterized angiosperm CYP79s (e.g. Andersen et al. 2000; Mikkelsen et al. 2000; Naur et al. 2003; Irmisch et al. 2013a, 2015). The high *K*_m_ values for l-phenylalanine (21.69 ± 6.26 mM) and l-tryptophan (24.15 ± 11.64 mM) indicate that these amino acids are likely not substrates in planta. This assumption is strengthened by the fact that the relative product formation for l-tyrosine (4918 ± 132 ng/h/ml assay) was ~250 times higher in comparison to that for l-tryptophan (23.3 ± 1.4 ng/h/ml assay) and l-phenylalanine (20.0 ± 1.2 ng/h/ml assay). To verify the in vitro data, full-length CYP79A118 and N-terminal truncated CYP79A118-M39 were transiently expressed in *Nicotiana benthamiana*. Leaves of transgenic plants were harvested 5 days after infiltration and aldoxime accumulation was analyzed using LC-MS. While plants expressing eGFP as negative control showed no aldoxime formation, expression of CYP79A118 and CYP79A118-M39 both resulted in the accumulation of *p*-hydroxyphenylacetaldoxime (See Fig. S2). Indole-3-acetaldoxime and phenylacetaldoxime, however, could not be detected in the transgenic plants, corroborating the kinetic parameters obtained in vitro.

### *CYP79A118* transcript abundance in different organs of *T. baccata*

**Fig. 5:**
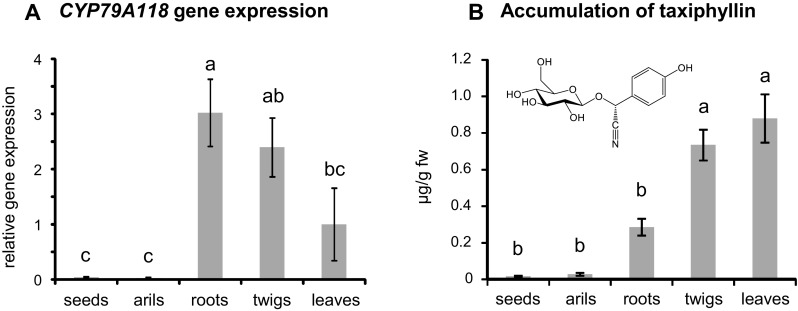
Transcript abundance of *CYP79A118* (**a**) and accumulation of taxiphyllin (**b**) in different organs of *T. baccata*. Gene expression was analyzed using qRT-PCR and taxiphyllin was measured using LC-MS/MS. Means and standard errors are shown (n = 7 biological replicates). A one-way ANOVA followed by a Tukey’s honest significance test was used to test for statistical significance. Different letters indicate significant differences between plant organs. *CYP79A118* gene expression: F = 8.039, *P* < 0.01; taxiphyllin accumulation: F = 30.16, *P* < 0.001)

Quantitative real-time PCR (qRT-PCR) was used to measure the transcript accumulation of *CYP79A118* in leaves, twigs, roots, seeds, and arils of *T. baccata* trees. To identify a reference gene with stable expression in the different organs, we tested the transcript accumulation of *actin* (Sun et al. 2013), 18S RNA (Ramirez-Estrada et al. 2016), and *GAPDH* (Zheng et al. 2016). Based on its low variability among different organs, *GAPDH* was chosen as a reference gene (See Fig. S3). qRT-PCR analysis of *CYP79A118* revealed transcript accumulation in roots, twigs, and leaves (Fig. 5a). In contrast, seeds and arils accumulated only trace levels of *CYP79A118* transcripts.

### Accumulation of taxiphyllin in different organs of *T. baccata*

Taxiphyllin has been described as the sole cyanogenic glycoside in leaves of different conifers, including *T. baccata* (Hegenauer 1959; Bijl-van Dijk et al. 1974). We used liquid chromatography-tandem mass spectrometry (LC-MS/MS) to quantify and compare the accumulation of taxiphyllin in the different plant organs of *T. baccata*. Highest amounts of taxiphyllin were found in leaves and twigs with 879 ± 131 ng/g fresh weight and 733 ± 84 ng/g fresh weight, respectively (Fig. 5b). Roots showed moderate accumulation (285 ± 46 ng/g fresh weight) while seeds and arils accumulated only trace amounts of taxiphyllin (Fig. 5b). Other cyanogenic glycosides such as prunasin, lotaustralin, linamarin, and amygdalin, which are commonly found in angiosperms, as well as the CYP79A118 product *p*-hydroxyphenylacetaldoxime could not be detected in the analyzed tissues.

## Discussion

### CYP79A118 is most likely involved in taxiphyllin formation

CYP79 enzymes play multiple roles in the biosynthesis of plant defense compounds and have been intensively investigated in angiosperms. However, whether these enzymes also occur in other plants such as gymnosperms and ferns has remained unclear. In this study, we report the identification of *CYP79A* genes in the transcriptomes of several conifer species. One of the genes, *CYP79A118* from *T. baccata*, was heterologously expressed in yeast and the recombinant enzyme produced *p*-hydroxyphenylacetaldoxime from l-tyrosine as substrate (Fig. 4). Although phenylalanine and tryptophan were also converted into aldoximes by recombinant CYP79A118 in vitro, the high *K*_m_ and low *V*_max_ values for both of these amino acids in comparison to those for tyrosine indicate that they are not employed as substrates in vivo. Indeed, transgenic *N. benthamiana* plants transiently expressing *CYP79A118* accumulated exclusively *p*-hydroxyphenylacetaldoxime and no other aldoximes (See Fig. S2). Several conifer species including *T. baccata* have been described to accumulate only a single cyanogenic glycoside, taxiphyllin, a compound derived from *p*-hydroxyphenylacetaldoxime (Towers et al. 1964; Bijl-van Dijk et al. 1974; Nahrstedt 1979). Considering its substrate specificity, it is conceivable that CYP79A118 catalyzes the first step in the formation of taxiphyllin in *T. baccata*. However, the genome of *T. baccata* is still not sequenced and thus we cannot exclude the presence of additional CYP79s that might provide *p*-hydroxyphenylacetaldoxime as precursor for taxiphyllin in this species instead or in addition to CYP79A118.

Taxiphyllin was found to accumulate in leaves, twigs, and roots, but not in seeds and arils (Fig. 5b). These findings confirm an older study, which reported that seeds and arils of *T. baccata* have no cyanide potential (Barnea et al. 1993). The accumulation of the cyanogenic glycoside dhurrin in sorghum has been shown to be regulated at the transcriptional level of CYP79A1 and CYP71E1, the first two enzymes of the dhurrin pathway (Busk and Moller 2002). Since *CYP79A118* is exclusively expressed in plant organs rich in taxiphyllin (Fig. 5), we hypothesize that it also controls cyanogenic glycoside accumulation in *T. baccata*. A possible co-regulation of taxiphyllin formation by CYP79A118 and the subsequent enzymatic step, the *p*-hydroxymandelonitrile-forming enzyme, as for dhurrin formation in sorghum *CYP79A1* and *CYP71E1* (Busk and Moller 2002), might allow the identification of the second step of taxiphyllin formation in *T. baccata*, probably catalyzed by a CYP71/736 homolog, via gene co-expression analysis.

The enzymes of the cyanogenic glycoside biosynthetic pathway in sorghum, CYP79A1, CYP71E1, UGT85B1, and a P450 reductase, are thought to be organized in membrane-associated protein complexes called metabolons, which promote channelling of the toxic and unstable intermediates and thus prevent autointoxication and loss of intermediates to side reactions (Nielsen et al. 2008; Moller 2010b; Jensen et al. 2011; Laursen et al. 2016). We could not detect aldoximes in our analyses of *T. baccata* trees and it is thus conceivable that the biosynthetic machinery for cyanogenic glycoside production in *Taxus* is also organized in protein complexes that do not allow the accumulation of free aldoximes.

### CYP79s represent an ancient P450 family in plants

CYP79s belong to a group of plant P450s that has been designated as CYP71 clan (Fig. 2). This multi-family clan, which comprises more than 37 CYP families, is believed to have evolved early in the evolution of the land plants (Nelson 2006; Nelson and Werck-Reichhart 2011). Until now, CYP79s were only known from angiosperms and were speculated to have arisen with the Magnoliids, a basal clade of the angiosperms (Nelson and Werck-Reichhart 2011; Hamberger and Bak 2013). The identification of *CYP79A* genes in the conifers now demonstrates that *CYP79* genes must have been present already in a common ancestor of the gymnosperms and angiosperms, which presumably existed in the middle of the Carboniferous period about 330 million years ago (Smith et al. 2010). Since some fern species are able to produce cyanogenic glycosides (Kofod and Eyjolfsson 1966, 1969), it is conceivable that they also possess *CYP79* genes for the production of the respective aldoxime precursors. Such a scenario would place the origin of CYP79s back in the early evolution of vascular plants about 430–400 million years ago (Smith et al. 2010).

Many angiosperms possess small *CYP79* gene families with an average number of four to five members (Irmisch et al. 2013a) and the encoded proteins are known to play diverse roles in plant defence. CYP79s, for instance, are not only the entry enzymes for cyanogenic glycoside formation, but can also produce aldoximes as precursors for other defence compounds such as glucosinolates and various phytoalexins (Halkier and Gershenzon 2006; Glawischnig 2007). Moreover, CYP79s are involved in herbivore-induced volatile formation (Irmisch et al. 2013a, 2014; Irmisch 2013b; McCormick et al. 2014; Luck et al. 2016) and they have been discussed to play a role in auxin homeostasis (Bak and Feyereisen 2001; Sugawara et al. 2009; Irmisch et al. 2015). In contrast, most of the conifer transcriptomes analysed in this study contained only a single *CYP79* gene, but the transcriptomes of the Podocarpaceae species *Phyllocladus hypophyllus* and the Taxaceae species *Amentotaxus argotaenia* and *Torreya taxifolia* contained two or three different *CYP79* gene fragments (See Table S2). Multiple *CYP79* gene copies in gymnosperm genomes might fulfil diverse biological roles comparable to those in angiosperms. However, the fact that ferns, conifers, and angiosperms all produce cyanogenic glycosides suggests an original function of CYP79s in the formation of these compounds.

## Methods

### Plant material

For the isolation of *CYP79A118, T. baccata* leaves were collected in September 2014 from a single tree growing near Ruhla, Thuringia, Germany (50°53′28.1"N 10°22′13.6"E). Plant material for cyanogenic glycoside analysis and qRT-PCR was collected in August 2016 from seven individual trees growing in Großeutersdorf, Thuringia, Germany (50°47′22.6"N 11°33′56.3"E).

### LC-MS/MS analysis of cyanogenic glycosides and aldoximes

Taxiphyllin was analyzed according to a previously described method for cyanogenic glycoside analysis (Irmisch et al. 2015). In brief, plant material was ground to powder in an ice-cold mortar. For extraction, 100 mg of frozen powder was transferred to a pre-cooled 2 ml microcentrifuge tube and 600 µl of ice-cold extraction solvent (80% aqueous methanol, gradient grade for liquid chromatography) were added. The tubes were immediately mixed until the powder was completely dispersed, and three chrome steel beads (4 mm diameter) were added to each tube. Plant tissues were subsequently extracted using a paint shaker (S0-10 M, Fluid Management, Wheeling, IL, USA) at 10 Hz for 4 min and cell debris was sedimented at 13,000 g for 30 min. The supernatant was stored at −20 °C before analysis.

Plant extracts were analyzed using liquid chromatography-tandem mass spectrometry (LC-MS/MS) performed on an Agilent 1200 HPLC system coupled to an API 5000 tandem mass spectrometer (Applied Biosystems, Darmstadt, Germany). Separation was achieved on a Zorbax Eclipse XDB-C18 column (50 × 4.6 mm, 1.8 µm, Agilent Technologies) with aqueous formic acid (0.2% v/v) and acetonitrile employed as mobile phases A and B, respectively. For the analysis of cyanogenic glycosides, the following elution profile was used: 0–0.5 min, 5% B in A; 0.5–6.0 min, 5–50% B; 6.0–7.5 min 100% B, and 7.5–10.5 min 5% B. The flow rate was set to 1.1 ml/min. The injection volume was 2 µl. The tandem mass spectrometer was operated in negative ionization mode (ionspray voltage, −4500 eV; turbo gas temp, 700 °C; nebulizing gas, 60 psi; curtain gas, 30 psi; heating gas, 50 psi; collision gas, 6 psi). The following mass analyzer settings were applied: collision energy (CE), −10 V; and declustering potential (DP), −15 V. MRM was used to monitor parent ion → product ion reactions for each analyte as follows: *m*/*z* 310.0 → 179.0 for taxiphyllin/dhurrin, *m*/*z* 294.0 → 89.0 (CE, −22; DP, −15) for prunasin, *m*/*z* 260.0 → 179.0 for lotaustralin, *m*/*z* 246.0 → 179.0 for linamarin, and *m*/*z* 456.0 → 179.0 for amygdalin. For quantification, the taxiphyllin enantiomer dhurrin was obtained from Sigma-Aldrich and used as external standard (See Fig. S4).

Aldoximes were measured from the same MeOH extracts using the same LC-MS/MS system as described above. Formic acid (0.2%) in water and acetonitrile were employed as mobile phases A and B, respectively, on a Zorbax Eclipse XDB-C18 column (50 × 4.6 mm, 1.8 µm). The elution profile was: 0–4 min, 10–70% B; 4–4.1 min, 70–100% B; 4.1–5 min 100% B, and 5.1–7 min 10% B at a flow rate of 1.1 ml/min. The tandem mass spectrometer was operated in positive ionization mode (ionspray voltage, 5500 eV; turbo gas temp, 700 °C; nebulizing gas, 60 psi; curtain gas, 30 psi; heating gas, 50 psi; collision gas, 6 psi). MRM was used to monitor precursor ion → product ion reactions for each analyte as follows: *m*/*z* 136.0 → 119.0 (collision energy (CE), 17 V; declustering potential (DP), 56 V) for phenylacetaldoxime; *m*/*z* 175.0 → 158.0 (CE, 17 V; DP, 56 V) for indole-3-acetaldoxime, *m*/*z* 102.0 → 69.0 (CE, 13 V; DP, 31 V) for 2-methylbutyraldoxime; *m*/*z* 102.0 → 46.0 (CE, 15 V; DP, 31 V) for 3-methylbutyraldoxime, and *m*/*z* 152.0 → 107.0 (CE, 27 V; DP, 100 V) for *p*-hydroxyphenylacetaldoxime. The concentration of aldoximes was determined using external standard curves made with authentic standards synthesized as described in the literature (Irmisch et al. 2013a).

### RNA extraction and reverse transcription

*Taxus baccata* plant material (leaves, roots, bark, arills, seeds) was flash-frozen in liquid nitrogen and stored at −80 °C until further processing. Total RNA was isolated from ground plant tissue using an InviTrap Spin Plant RNA kit (Stratec, Berlin, Germany) according to manufacturer’s instructions. RNA concentration and purity were assessed using a spectrophotometer (NanoDrop 2000c, Thermo Scientific, Wilmington, DE, USA). RNA was treated with TurboDNase (ThermoFisher Scientific, https://www.thermofisher.com) prior to cDNA synthesis. Single-stranded cDNA was prepared from 1 µg of DNase-treated RNA using SuperScript™ III reverse transcriptase and oligo (dT_12–18_) primers (Invitrogen, Carlsbad, CA, USA).

### Identification of *CYP79* genes in conifers

Transcriptomes of 72 different conifer species (See Table S1) were downloaded from OneKP (https://sites.google.com/a/ualberta.ca/onekp/). The annotated protein sequences (Jia and Chen 2016) were searched for homologs of angiosperm CYP79s using BLASTP. At a combined threshold of E-value (10^−20^) and identity (38%), 2134 putative sequences were identified. In view of high sequence similarity shared by CYP79s and other P450 families, such as CYP703 family, a phylogenetic analysis with P450 sequences from different families was used to remove non-CYP79 sequences. The full-length genes identified were designated as *CYP79A117* (*M. glyptostroboides*), *CYP79A118* (*T. baccata*), *CYP79A119* (*Dacrycarpus compactus*), *CYP79A120* (*Dacrydium balansae*), *CYP79A121* (*Falcatifolium taxoides*), *CYP79A122* (*A. argotaenia*), and *CYP79A123* (*T. taxifolia*) according to the general P450 nomenclature (D.R. Nelson, P450 Nomenclature Committee). Some of these genes may be orthologous, but transcriptomes do not provide synteny data to verify this. The complete open reading frame of *CYP79A118* was amplified from cDNA obtained from *T. baccata* leaves. The PCR product was cloned into the sequencing vector pCR^®^-Blunt II-TOPO^®^ (Invitrogen) and both strands were fully sequenced using the Sanger method. Primer sequence information is given in Table S3.

### Heterologous expression of *CYP79A118* in yeast

For heterologous expression in *Saccharomyces cerevisiae*, the complete open reading frame of *CYP79A118* and the 5′ truncated version *CYP79A118-M39* were cloned into the pESC-Leu2d vector (Ro et al. 2008) as *No*tI/*Sac*I fragments. The resulting constructs were transferred into the *S. cerevisiae* strain WAT11 (Pompon et al. 1996) and single yeast colonies were picked to inoculate starting cultures containing 30 ml SC minimal medium lacking leucine (6.7 g/l yeast nitrogen base without amino acids, but with ammonium sulfate). Other components: 100 mg/l of l-adenine, l-arginine, l-cysteine, l-lysine, l-threonine, l-tryptophan and uracil; 50 mg/l of the amino acids l-aspartic acid, l-histidine, l-isoleucine, l-methionine, l-phenylalanine, l-proline, l-serine, l-tyrosine, l-valine; 20 g/l d-glucose. The cultures were grown overnight at 28 °C and 180 rpm. One OD of the starting cultures (approx. 2 × 10^7^ cells/ml) was used to inoculate 100 ml YPGA full medium cultures (10 g/l yeast extract, 20 g/l bactopeptone, 74 mg/l adenine hemisulfate, 20 g/l d-glucose) which were grown for 32–35 h (until OD about five), induced by the addition of galactose and cultured for another 15–18 h. The cultures were centrifuged (7500 g, 10 min, 4 °C), the supernatant was decanted, and the cell pellets were resuspended in 30 ml TEK buffer (50 mM Tris–HCl pH 7.5, 1 mM EDTA, 100 mM KCl) and centrifuged again. Then, the pellets were carefully resuspended in 2 ml of TES buffer (50 mM Tris–HCl pH 7.5, 1 mM EDTA, 600 mM sorbitol, 10 g/l bovine serum fraction V protein and 1.5 mM β-mercaptoethanol) and transferred to a 50 ml conical tube. Glass beads (0.45–0.50 mm diameter, Sigma–Aldrich Chemicals, Steinheim, Germany) were added so that they filled the full volume of the cell suspension. Yeast cell walls were disrupted by five cycles of 1 min shaking by hand and subsequent cooling down on ice for 1 min. The crude extracts were recovered by washing the glass beads four times with 5 ml TES. The combined washes were centrifuged (7500 g, 10 min, 4 °C), and the supernatant was transferred to another tube and centrifuged again (100,000 g, 60 min, 4 °C). The resulting microsomal protein fractions were homogenized in 2 ml TEG buffer (50 mM Tris–HCl, 1 mM EDTA, 30% w/v glycerol) using a glass homogenizer (Potter–Elvehjem, Fisher Scientific, Schwerte, Germany). Aliquots were stored at −20 °C.

### Analysis of recombinant CYP79A118

To determine the substrate specificity of full-length and N-terminal truncated CYP79A118, yeast microsomes harboring recombinant protein were incubated for 60 min at 25 °C and 300 rpm individually with the potential substrates l-Phe, l-Val, l-Leu, l-Ile, l-Tyr, and l-Trp in glass vials containing 300 µl of the reaction mixture (75 mM sodium phosphate buffer (pH 7.0), 1 mM substrate (concentration was variable for K_*m*_ determination), 1 mM NADPH, and 10 µl of the prepared microsomes). Reaction products where analyzed using LC-MS/MS as described below.

For the determination of the *K*_m_ values, assays were carried out as triplicates and stopped by placing on ice after 300 µl MeOH were added. Enzyme concentrations and incubation times were chosen so that the reaction velocity was linear during the incubation time period.

### Transient expression of CYP79A118 and CYP79A118-M39 in* N. benthamiana*

For gene expression in *N. benthamiana*, the complete ORF as well as the N-terminal truncated ORF (M39) of *CYP79A118* were cloned into the pCAMBiA2300U vector. Resulting vectors carrying either one of the *CYP79A118* constructs, *eGFP*, or the construct pBIN::*p19* were separately transferred into *Agrobacterium tumefaciens* strain LBA4404. Five milliliter of an overnight culture (220 rpm, 28 °C) were used to inoculate 50 ml LB medium (50 µg/ml kanamycin, 25 µg/ml rifampicin and 25 µg/ml gentamicin) for overnight growth. The following day, the cultures were centrifuged (4000 g, 5 min) and the cells were resuspended in infiltration buffer (10 mM MES, 10 mM MgCl_2_, 100 µM acetosyringone, pH 5.6) to reach a final OD of 0.5. After shaking for at least 1 h at room temperature, the cultures carrying *CYP79A118, CYP79A118-M39*, or *eGFP* were mixed with an equal volume of cultures carrying pBIN::*p19*.

For transformation, 3–4 week-old *N. benthamiana* plants were dipped upside down in an *A. tumefaciens* solution and vacuum was applied to infiltrate the leaves. Infiltrated plants were shaded with cotton tissue to protect them from direct irradiation. Five days after transformation, CYP79A118 products were extracted with methanol from grinded leaves and measured using LC-MS/MS as described above.

### qRT-PCR analysis

cDNA was prepared as described above and diluted 1:10 with water. qPCR primers for *CYP79A118* were designed having a Tm ≥ 60 °C, a GC content between 50–58%, and a primer length of 21 nucleotides (Table S3). The amplicon size was 108 bp. The specificity and efficiency of the primers were confirmed by agarose gel electrophoresis, melting curve analysis, standard curve analysis, and by sequence verification of cloned PCR amplicons. *Glyceraldehyde 3-phosphate dehydrogenase* (*GAPDH*) was used as a reference gene (Zheng et al. 2016). Samples were run in triplicate using Brilliant III Ultra-Fast SYBR^®^ Green QPCR Master Mix (Stratagene, Carlsbad, CA, USA). The following PCR conditions were applied for all reactions: Initial incubation at 95 °C for 3 min followed by 40 cycles of amplification (95 °C for 5 s, 60 °C for 10 s). Plate reads were taken at the end of the extension step of each cycle. Data for the melting curves were recorded at the end of cycling from 60 to 95 °C.

All samples were run on the same PCR machine (Bio-Rad CFX Manager 3.1, Bio-Rad Laboratory, Hercules, CA, USA) in an optical 96-well plate. For each tissue, seven biological replicates were analyzed in triplicate.

### Sequence analysis and phylogenetic tree construction

An alignment of conifer *CYP79* genes and characterized *CYP* genes from angiosperms was constructed using the MUSCLE (codon) algorithm (gap open, −2.9; gap extend, 0; hydrophobicity multiplier, 1.2; clustering method, UPGMB) implemented in MEGA6 (Tamura et al. 2011). Tree reconstruction was done with MEGA6 using a maximum likelihood algorithm (model/method, General Time Reversible model; substitutions type, nucleotide; rates among sites, gamma distributed (G); gamma parameters, 5; gaps/missing data treatment, partial deletion; site coverage cutoff, 90%). A bootstrap resampling analysis with 1000 replicates was performed to evaluate the tree topology.

### Statistical analysis

Differences in taxiphyllin content and relative expression of *CYP79A118* in the tested organs of *T. baccata* trees were analyzed with one-way ANOVAs. Pairwise comparisons were performed with Tukey’s honest significance test (α = 0.05) using the agricolae package in R (R Core Team 2014; de Mendiburu et al. 2014). Data are presented as means ± SE.

## Electronic supplementary material

Below is the link to the electronic supplementary material.

Supplementary material 1 (DOCX 26 KB)

Supplementary material 2 (PPTX 583 KB)

## Abbreviations

CYP: Cytochrome P450 monooxygenase
qRT-PCR: Quantitative real-time PCR
LC-MS/MS: Liquid chromatography-tandem mass spectrometry
