# Supplementary material for: CYP79 P450 monooxygenases in gymnosperms: CYP79A118 is associated with the formation of taxiphyllin in Taxus baccata
Source: Plant Mol Biol. 2017 Aug 9;95(1):169–80. doi: 10.1007/s11103-017-0646-0 (PMC5594043; doi:10.1007/s11103-017-0646-0)
Supplement: Supplementary file 1 — Supplementary material 1 (DOCX 26 KB) [file 11103_2017_646_MOESM1_ESM.docx]

**Table S1**

**The 72 conifer species screened in this study.**

| **Species** | **OneKP Transcriptome Identifiers** |
| --- | --- |
| *Abies lasiocarpa*  *Acmopyle pancheri*  *Agathis robusta*  *Amentotaxus argotaenia*  *Araucaria rulei*  *Arucaria sp.*  *Athrotaxis cupressoides*  *Austrocedrus chilensis*  *Austrotaxus spicata*  *Callitris gracilis*  *Callitris macleayana*  *Calocedrus decurrens*  *Cathaya agryrophylla*  *Cedrus libani*  *Cephalotaxus harringtonia*  *Chamaecyparis lawsoniana*  *Cryptomeria japonica*  *Cunninghamia lanceolata*  *Cupressus dupreziana*  *Dacrycarpus compactus*  *Dacrydium balansae*  *Diselma archeri*  *Falcatifolium taxoides*  *Fokienia hodginsii*  *Glyptostrobus pensilis*  *Halocarpus bidwillii*  *Juniperus scopulorum*  *Keteleeria evelyniana*  *Lagarostrobos franklinii*  *Larix speciosa*  *Manoao colensoi*  *Metasequoia glyptostroboides*  *Microbiota decussata*  *Microcachrys tetragona*  *Microstrobos fitzgeraldii*  *Nageia nagi*  *Neocallitropsis pancheri*  *Nothotsuga longibracteata*  *Papuacedrus papuana*  *Parasitaxus usta*  *Phyllocladus hypohyllus*  *Picea engelmanii*  *Pilgerodendron uviferum*  *Pinus jeffreyi*  *Pinus parviflora*  *Pinus ponderosa*  *Pinus radiata*  *Platycladus orientalis*  *Podocarpus coriaceus*  *Podocarpus rubens*  *Prumnopitys andina*  *Pseudolarix amabilis*  *Pseudotaxus chienii*  *Pseudotsuga menziesii*  *Retrophyllum minus*  *Saxegothaea conspicua*  *Sciadopitys verticillata*  *Sequoia sempervirens*  *Sequoiadendron giganteum*  *Sundacarpus amarus*  *Taiwania cryptomerioides*  *Taxodium distichum*  *Taxus baccata*  *Taxus cuspidata*  *Tetraclinis sp.*  *Thuja plicata*  *Thujopsis dolabrata*  *Torreya nucifera*  *Torreya taxifolia*  *Tsuga heterophylla*  *Widdringtonia cedarbergensis*  *Wollemia nobilis* | VSRH  HILW  MIXZ  IAJW  XTZO  ACWS  XIRK  YYPE  BTTS  IFLI  RMMV  FRPM  NPRL  GGEA  NVGZ  AIGO  GMHZ  ZQVF  QNGJ  FMWZ  IZGN  GKCZ  PLYX  UEVI  OXGJ  OWFC  XMGP  JUWL  ZQWM  WVWN  CDFR  NRXL  XQSG  MHGD  BBDD  UUJS  JDQB  AREG  OVIJ  JZVE  JRNA  AWQB  ETCJ  MFTM  IIOL  JBND  DZQM  BUWV  SCEB  XLGK  EGLZ  AQFM  YLPM  IOVS  VGSX  QCGM  YFZK  HBGV  QFAE  KLGF  QSNJ  FHST  WWSS  ZYAX  CGDN  VFYZ  NKIN  HQOM  EFMS  GAMH  AUDE  RSCE |

**Table S2**

***CYP79A* genes and gene fragments identified in this study.** The numbers in brackets given in the ‘comments’ column indicate the length of the encoded proteins, and the four-letter codes in the ‘Species’ column are OneKP transcriptome identifiers.

| **Species** | **Family** | **Designation** | **Comments** |
| --- | --- | --- | --- |
| *Phyllocladus hypophyllus* (JRNA)  *Phyllocladus hypophyllus* (JRNA)  *Dacrydium balansae* (IZGN)  *Falcatifolium taxoides* (PLYX)  *Dacrycarpus compactus* (FMWZ)  *Podocarpus coriacens* (SCEB)  *Podocarpus rubens* (XLGK)  *Cephalotaxus harringtonia* (NVGZ)  *Amentotaxus argotaenia* (IAJW)  *Torreya taxifolia* (EFMS)  *Taxus baccata* (WWSS)  *Metasequoia glyptostroboides* (NRXL) | Podocarpaceae  Podocarpaceae  Podocarpaceae  Podocarpaceae  Podocarpaceae  Podocarpaceae  Podocarpaceae  Taxaceae  Taxaceae  Taxaceae  Taxaceae  Cupressaceae | *CYP79A120*  *CYP79A121*  *CYP79A119*  *CYP79A122*  *CYP79A123*  *CYP79A118*  *CYP79A117* | fragment (380 AA)  fragment (446 AA)  full-length (537 AA)  full-length (540 AA)  full-length (533 AA)  fragment (389 AA)  fragment (338 AA)  fragment (487 AA)  full-length (537 AA)  full-length (519 AA)  full-length (582 AA)  full-length (557 AA) |

**Table S3**

**Oligonucleotides used for isolation and qRT-PCR analysis of conifer *CYP79* genes.**

| **Name** | **Sequence** | | **Usage** |
| --- | --- | --- | --- |
| WWSS9882-fwd  WWSS9882-rev  WWSS-NotI_fwd_WT  WWSS-NotI_fwd_M38  WWSS-SacI_rev  TbCyp79_fwd3  TbCyp79_rev3  TbGAPDH_fwd1  TbGAPDH_rev1  TbActin_fwd1  TbActin_rev1  Tb18S-RNA_fwd1  Tb18S-RNA_rev1 | | ATGGTGTGGGGATTTGTTTTC  CTATGGATAGAGATGGTTGGGA  CTTTGCGGCCGCAATGGTGTGGGGATTTGTTTTC  GTTAGCGGCCGCAATGCGCCATCATCTTCATATTG  GGGTGAGCTCCTATGGATAGAGATGGTTGGG  TGTCAATGGAGGACACCACAG  GAGGATCCTCCCACACACATG  CGGAGACAGTCGATCAAGC  CCCATCCTCAACCCAATAA  AAGAGAAGCTTGCTTATGTAGC  TCTGATATCCACATCACACTTC  GTGCACAAAATCCCGACTCT  GCGATCCGTCGAGTTATCAT | ORF cloning  ORF cloning  expression M1  expression M38  expression  qRT-PCR  qRT-PCR  qRT-PCR  qRT-PCR  qRT-PCR  qRT-PCR  qRT-PCR  qRT-PCR |
