# Supplementary material for: CYP79 P450 monooxygenases in gymnosperms: CYP79A118 is associated with the formation of taxiphyllin in Taxus baccata
Source: Plant Mol Biol. 2017 Aug 9;95(1):169–80. doi: 10.1007/s11103-017-0646-0 (PMC5594043; doi:10.1007/s11103-017-0646-0)
Supplement: Supplementary file 2 — Supplementary material 2 (PPTX 583 KB) [file 11103_2017_646_MOESM2_ESM.pptx]

## Slide 1
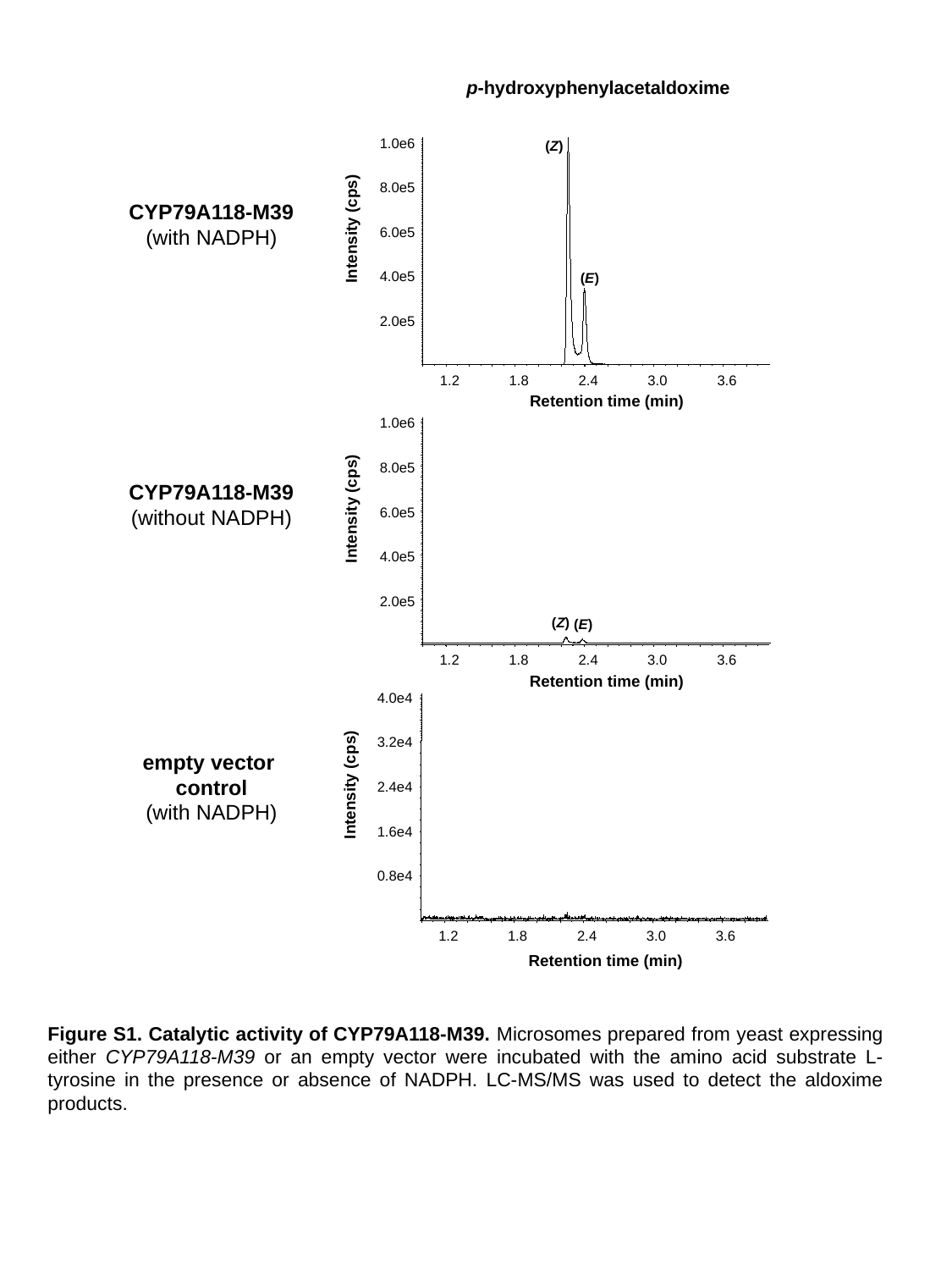

p-hydroxyphenylacetaldoxime
(Z)
1.0e6
8.0e5
Intensity (cps)
6.0e5
4.0e5
2.0e5
1.2
1.8
2.4
3.0
3.6
Retention time (min)
CYP79A118-M39
(with NADPH)
(E)
1.0e6
8.0e5
Intensity (cps)
6.0e5
4.0e5
2.0e5
1.2
1.8
2.4
3.0
3.6
Retention time (min)
CYP79A118-M39
(without NADPH)
(Z)
(E)
4.0e4
3.2e4
Intensity (cps)
2.4e4
1.6e4
0.8e4
1.2
1.8
2.4
3.0
3.6
Retention time (min)
empty vector
control
(with NADPH)
Figure S1. Catalytic activity of CYP79A118-M39. Microsomes prepared from yeast expressing either CYP79A118-M39 or an empty vector were incubated with the amino acid substrate L-tyrosine in the presence or absence of NADPH. LC-MS/MS was used to detect the aldoxime products.

## Slide 2
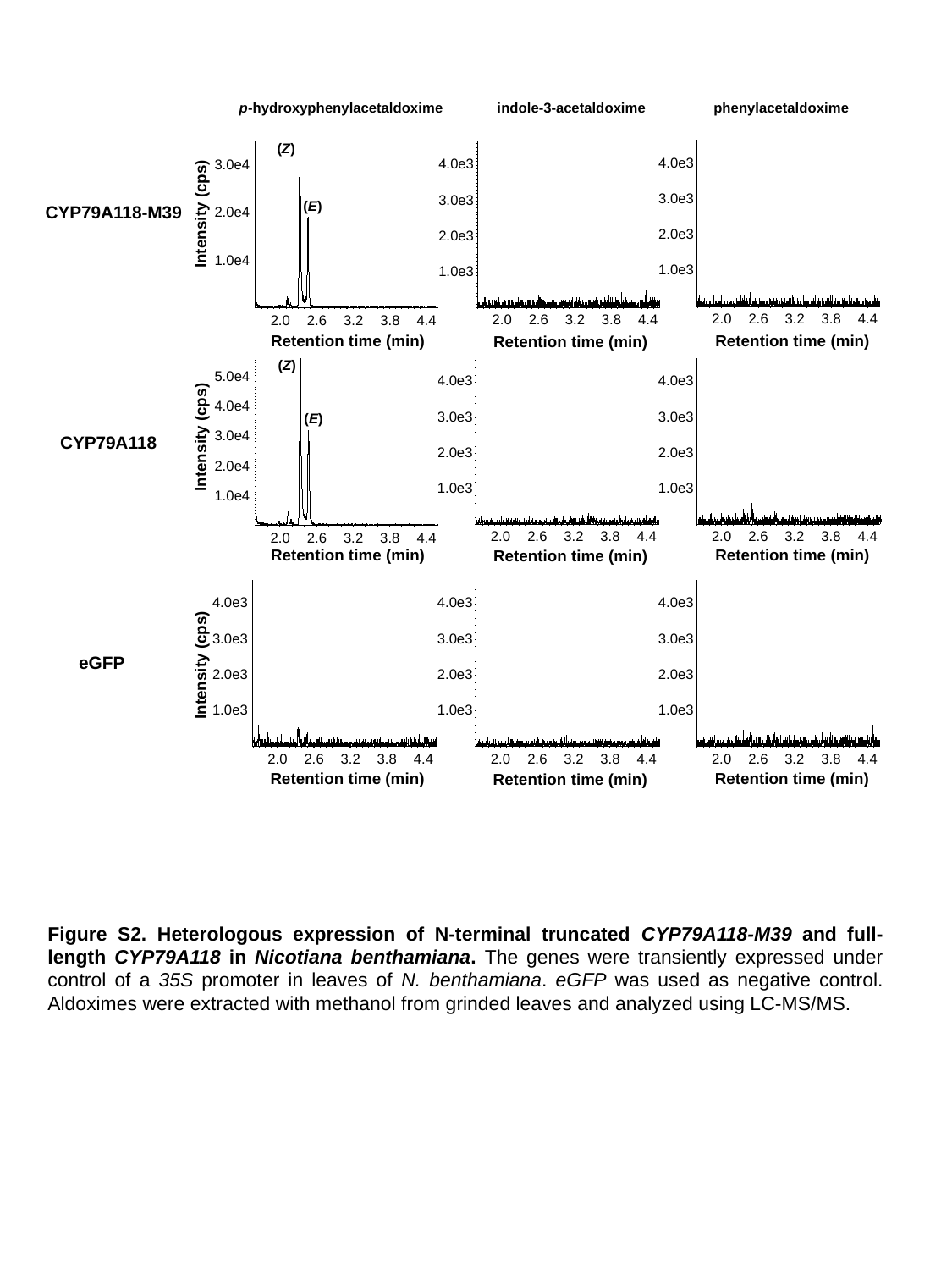

p-hydroxyphenylacetaldoxime
indole-3-acetaldoxime
phenylacetaldoxime
(Z)
3.0e4
(E)
2.0e4
Intensity (cps)
1.0e4
2.0
2.6
3.2
3.8
4.4
Retention time (min)
4.0e3
3.0e3
2.0e3
1.0e3
2.0
2.6
3.2
3.8
4.4
4.0e3
3.0e3
2.0e3
1.0e3
2.0
2.6
3.2
3.8
4.4
Retention time (min)
CYP79A118-M39
Retention time (min)
(Z)
5.0e4
4.0e4
(E)
3.0e4
Intensity (cps)
2.0e4
1.0e4
2.0
2.6
3.2
3.8
4.4
Retention time (min)
4.0e3
3.0e3
2.0e3
1.0e3
2.0
2.6
3.2
3.8
4.4
4.0e3
3.0e3
2.0e3
1.0e3
2.0
2.6
3.2
3.8
4.4
CYP79A118
Retention time (min)
Retention time (min)
4.0e3
3.0e3
2.0e3
1.0e3
2.0
2.6
3.2
3.8
4.4
4.0e3
3.0e3
2.0e3
1.0e3
2.0
2.6
3.2
3.8
4.4
4.0e3
3.0e3
2.0e3
1.0e3
2.0
2.6
3.2
3.8
4.4
eGFP
Intensity (cps)
Retention time (min)
Retention time (min)
Retention time (min)
Figure S2. Heterologous expression of N-terminal truncated CYP79A118-M39 and full-length CYP79A118 in Nicotiana benthamiana. The genes were transiently expressed under control of a 35S promoter in leaves of N. benthamiana. eGFP was used as negative control. Aldoximes were extracted with methanol from grinded leaves and analyzed using LC-MS/MS.

## Slide 3
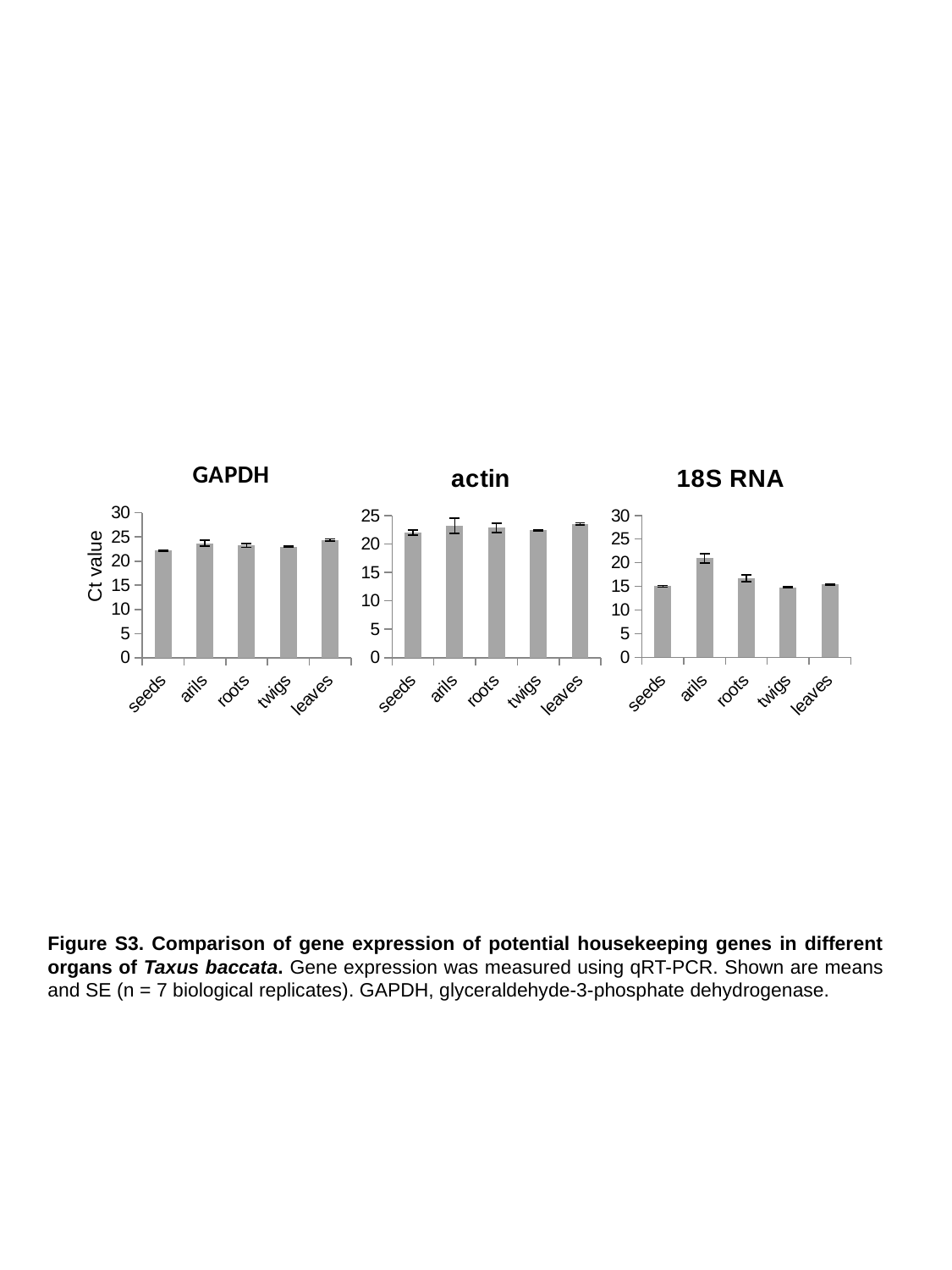

### Chart:
| Category | GAPDH |
|---|---|
| seeds | 22.179456207528343 |
| arils | 23.719908578755724 |
| roots | 23.231207655152282 |
| twigs | 23.012300110771452 |
| leaves | 24.378710719568485 |
### Chart:
| Category | actin |
|---|---|
| seeds | 21.995796241755187 |
| arils | 23.21507181228407 |
| roots | 22.824714026756137 |
| twigs | 22.414565562113015 |
| leaves | 23.519696916242182 |
### Chart: 18S RNA
| Category | 18S RNA |
|---|---|
| seeds | 15.035316289959663 |
| arils | 20.93900133147491 |
| roots | 16.725605759871232 |
| twigs | 14.790123320474311 |
| leaves | 15.386167330486256 |Ct value
Figure S3. Comparison of gene expression of potential housekeeping genes in different organs of Taxus baccata. Gene expression was measured using qRT-PCR. Shown are means and SE (n = 7 biological replicates). GAPDH, glyceraldehyde-3-phosphate dehydrogenase.

## Slide 4
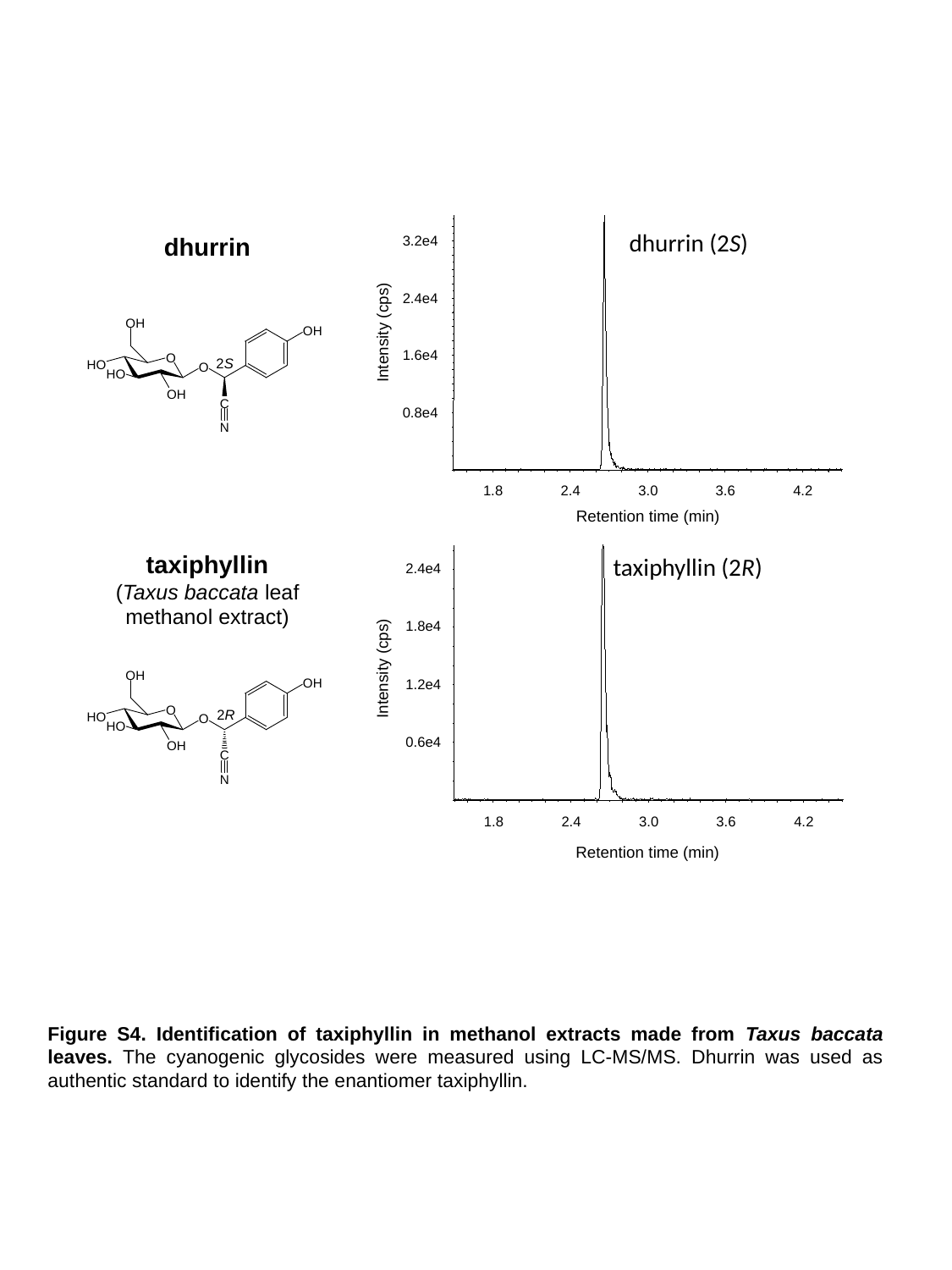

dhurrin (2S)
3.2e4
2.4e4
1.6e4
0.8e4
1.8
2.4
3.0
3.6
4.2
dhurrin
Intensity (cps)
2S
Retention time (min)
taxiphyllin
(Taxus baccata leaf
methanol extract)
taxiphyllin (2R)
2.4e4
1.8e4
1.2e4
0.6e4
1.8
2.4
3.0
3.6
4.2
Intensity (cps)
2R
Retention time (min)
Figure S4. Identification of taxiphyllin in methanol extracts made from Taxus baccata leaves. The cyanogenic glycosides were measured using LC-MS/MS. Dhurrin was used as authentic standard to identify the enantiomer taxiphyllin.
